# Cost-effectiveness analysis of protease inhibitor monotherapy vs. ongoing triple-therapy in the long-term management of HIV patients

**DOI:** 10.7448/IAS.17.4.19498

**Published:** 2014-11-02

**Authors:** Lars Oddershede, Simon Walker, Nicholas Paton, Wolfgang Stöhr, David Dunn, Mark Sculpher

**Affiliations:** 1 Danish Center for Healthcare Improvements, Aalborg University, Aalborg East, Denmark; 2 Centre for Health Economics, The University of York, York, UK; 3 MRC Clinical Trials Unit, UCL, London, UK

## Abstract

**Introduction:**

Protease inhibitors might be sufficient to maintain complete virological suppression when used as monotherapy for HIV-1-positive patients who have achieved sustained virological suppression on combination antiretroviral therapy (ART). The present study estimated the cost-effectiveness of a strategy of switching the ART to protease inhibitor monotherapy (PIM) with prompt return to combination therapy in the event of viral load rebound compared to continuing the ongoing triple-therapy (OTT) in the long-term management of HIV-1-positive patients.

**Materials and Methods:**

A within-trial cost-effectiveness analysis and modelling of lifetime cost-effectiveness was performed based on a randomized controlled trial of Protease Inhibitor monotherapy Versus Ongoing Triple-therapy (PIVOT). The setting was HIV outpatient care in the UK National Health Service, and the trial involved 587 patients, aged 18 years or more, who achieved sustained virological suppression and have a CD4+ cell count >100 cells/mm^3^. Outcomes were NHS costs (2012 UK pounds sterling) and quality-adjusted life-years (QALY) with comparative results presented as incremental cost-effectiveness ratios (ICERs).

**Results:**

Overall, PIM was cost-effective compared to OTT. PIM was cost-saving due to large savings in the ART drug costs while being no less effective in terms of QALYs in the within-trial analysis and only marginally less effective with modelling. In the base-case within-trial analysis, the incremental total cost per patient was −£6,424.11 (95% confidence interval:−£7,418.84 to −£5,429.38) and the incremental QALY was 0.0051 (95% confidence interval: −0.0479 to 0.0582) making PIM dominant compared to OTT. Multiple sensitivity analyses were conducted to assess the importance of assumptions surrounding drug costs, missing data, trial protocol driven costs and mortality. In all sensitivity analyses, PIM was cost-saving and no marked difference in QALY was observed. Modelling of life time costs and QALYs showed significant cost-savings and marginally less effectiveness such that switching to PIM appeared cost-effective at accepted cost-effectiveness thresholds.

**Conclusions:**

The results suggest that PIM is a cost-effective treatment strategy compared to OTT for HIV-1-positive patients who have achieved sustained virological suppression.

